# Host-Defense Peptides with Therapeutic Potential from Skin Secretions of Frogs from the Family Pipidae

**DOI:** 10.3390/ph7010058

**Published:** 2014-01-15

**Authors:** J. Michael Conlon, Milena Mechkarska

**Affiliations:** Department of Biochemistry, College of Medicine and Health Sciences, United Arab Emirates University, Al Ain 17666, UAE; E-Mail: mpanteva@uaeu.ac.ae

**Keywords:** frog skin, magainin, PGLa, caerulein-precursor fragment, xenopsin-precursor-fragment, hymenochirin

## Abstract

Skin secretions from frogs belonging to the genera *Xenopus*, *Silurana*, *Hymenochirus*, and *Pseudhymenochirus* in the family Pipidae are a rich source of host-defense peptides with varying degrees of antimicrobial activities and cytotoxicities to mammalian cells. Magainin, peptide glycine-leucine-amide (PGLa), caerulein-precursor fragment (CPF), and xenopsin-precursor fragment (XPF) peptides have been isolated from norepinephrine-stimulated skin secretions from several species of *Xenopus* and *Silurana*. Hymenochirins and pseudhymenochirins have been isolated from *Hymenochirus boettgeri* and *Pseudhymenochirus merlini*. A major obstacle to the development of these peptides as anti-infective agents is their hemolytic activities against human erythrocytes. Analogs of the magainins, CPF peptides and hymenochirin-1B with increased antimicrobial potencies and low cytotoxicities have been developed that are active (MIC < 5 μM) against multidrug-resistant clinical isolates of *Staphylococcus aureus*, *Escherichia coli*, *Acinetobacter baumannii*, *Stenotrophomonas maltophilia* and *Klebsiella pneumoniae*. Despite this, the therapeutic potential of frog skin peptides as anti-infective agents has not been realized so that alternative clinical applications as anti-cancer, anti-viral, anti-diabetic, or immunomodulatory drugs are being explored.

## 1. Introduction

The emergence in all regions of the World of strains of pathogenic bacteria and fungi with resistance to commonly used antibiotics constitutes a serious threat to public health and has necessitated a search for novel types of antimicrobial agent to which the microorganisms have not been exposed. Although effective new types of antibiotics against multidrug-resistant Gram-positive bacteria such as methicillin-resistant *Staphylococcus aureus* (MRSA) have been introduced or are in clinical trials, the situation regarding new treatment options for infections produced by multidrug-resistant Gram-negative pathogens such as *Acinetobacter* *baumannii*, *Pseudomonas aeruginosa*, *Klebsiella pneumoniae*, and *Stenotrophomonas maltophilia* is less encouraging [1]. There is an urgent need for new types of antimicrobial agents with activity against these microorganisms that also possess appropriate pharmacokinetic and toxicological profiles.

Peptides with potent antibacterial and antifungal activity play an important role in the system of innate immunity that predates adaptive immunity and constitutes the first-line defense against invading pathogens for a wide range of vertebrate and invertebrate species. Skin secretions from many species of Anura (frogs and toads) contain cytotoxic peptides, often in very high concentrations, with broad-spectrum antibacterial and antifungal activities and the ability to permeabilize mammalian cells [2,3]. Although usually referred to as antimicrobial peptides, these components are multifunctional, displaying cytokine-mediated immunomodulatory properties as well as anti-cancer, anti-viral, chemoattractive, and insulin-releasing activities. Consequently, it is more informative, therefore, to refer to them as host-defense peptides rather than as exclusively antimicrobial peptides [4]. It is a common fallacy that all anurans produce host-defense peptides in their skin secretions. At the time of writing peptides with antimicrobial activity have been identified in the skins of frogs from species belonging to the Alytidae, Bombinatoridae, Hylidae, Hyperoliidae, Leiopelmatidae, Leptodactylidae, Myobatrachidae, Pipidae, and Ranidae families [2,3]. The sporadic species distribution suggests that production of cytotoxic peptides in the skin may confer some evolutionary advantage to the organism, but is not necessary for survival. It has been suggested that cutaneous symbiotic bacteria may provide the major system of defense against pathogenic microorganisms in the environment with antimicrobial peptides assuming a supplementary role in some species [2]. In the laboratory or in the field, mild electrical stimulation or injections of norepinephrine into the dorsal sac are effective methods of inducing secretion of skin peptides that do not appear to cause harm or undue distress to the animal [5].

Frog skin host-defense peptides vary in size from as small as eight up to 63 amino acid residues. A comparison of their amino acid sequences reveals the lack of any conserved domains that are associated with biological activity. However, with few exceptions, the peptides are cationic, generally with a charge of between +2 and +6 at pH 7 due to the presence of multiple lysine residues, and contain about 50% hydrophobic amino acids. At the time of writing, the Antimicrobial Peptide Database (http://aps.unmc.edu/AP) lists 929 amphibian host-defense peptides, 96% of which have a charge of between +1 and +6 and 90% have between 40% and 70% hydrophobic residues. Circular dichroism and NMR studies have shown that they generally lack stable secondary structure in aqueous solution, but have the propensity to form an amphipathic α-helix in the environment of a phospholipid vesicle or in a membrane-mimetic solvent such as 50% trifluoroethanol-water [2,3]. There is no single mechanism by which peptides produce cell death, but their action does not involve binding to a specific receptor rather a non-specific interaction with the bacterial cell membrane that results in permeabilization and ultimate disintegration [6,7]. Consequently, the frog skin peptides are usually active against microorganisms that are resistant to currently licensed antibiotics due to their markedly different and highly destructive mode of action.

The frog skin host-defense peptides may be grouped together in sets or families on the basis of limited similarities in amino acid sequence. Skin secretions from a single species frequently contain several members of a particular peptide family that are presumed to have arisen from multiple duplications of an ancestral gene. The molecular heterogeneity of the peptides within a particular family is considerable and this variation in primary structure is reflected in a wide variability in antimicrobial potencies and specificities for different microorganisms. It has been suggested that this multiplicity may provide a broader spectrum of defense against the range of pathogenic microorganisms encountered in the environment [8] but conclusive evidence to support this assertion is still required.

A major obstacle to the development of frog skin peptides as therapeutically valuable anti-infective agents, particularly if they are to be administered systemically, is their varying degrees of cytotoxicity to mammalian cells and their short-lives in the circulation. However, effective strategies have been developed to design analogs of the naturally occurring peptides that maintain or increase antimicrobial potency while displaying reduced cytotoxicity to human cells, such as erythrocytes [9,10,11]. Peptides administered to infected skin or skin lesions can penetrate into the *stratum corneum* to kill microorganisms so that future therapeutic applications are more likely to involve topical rather than systemic administration. This review will examine possible clinical application of well characterized peptides that have been isolated from skin secretions from African clawed frogs belonging to the family Pipidae together with analogs of the naturally occurring peptides that show improved therapeutic potential.

## 2. The Family Pipidae

The Pipidae are the only principally aquatic group of frogs and, at this time, the taxon comprises 33 well characterized species distributed in five genera: *Hymenochirus*, *Pipa*, *Pseudhymenochirus*, *Silurana*, and *Xenopus* [12]. All are found in Africa south of the Sahara, except for members of the genus *Pipa* which are found in South America. Pipidae is sister-group to Rhinophrynidae (represented by a single species, the Mexican burrowing toad *Rhinophrynus dorsalis*) and the two families are united in the Pipoidea [13]. Phylogenetic relationships within the Pipidae are not entirely clear. Molecular analyses based upon the comparison of the nucleotide sequences of mitochondrial [14,15,16] and multiple nuclear [17] genes strongly support sister-group relationships between *Silurana* and *Xenopus*, united in the monophyletic clade Xenopodinae. Molecular data also provide support for *Pipa* as sister-group to all other extant pipids [16,18,19]. The origin of the Pipidae is at least Late Jurassic (150 MYA) and it is suggested that the breakup of Gondwanaland led to the establishment of *Pipa* in South America and the remaining genera (*Xenopus* + *Silurana* + *Hymenochirus* + *Pseudhymenochirus*) in Africa [17,20].

The clawed frogs of the genus *Xenopus* currently comprise 19 well characterized species although several unnamed species have been reported [12]. The genus has a complex evolutionary history involving both bifurcating and reticulating modes of speciation [14,15]. Allopolyploidization events, in which two species hybridize and the descendant inherits the complete genome of both ancestors, have given rise to tetraploid, octoploid, and dodecaploid species with no extant *Xenopus* species retaining the diploid status that is thought to be related to the ancestral state existing prior to one or more whole genome duplications. At this time, the ten tetraploid *Xenopus* species have been divided into three species groups on the basis of similarities in morphology, advertisement calls, and/or nucleotide sequences of mitochondrial genes: the *laevis* group includes *X. laevis*, *X. gilli*, *X. largeni*, *X. petersii*, and *X. victorianus*; the *muelleri* group includes *X. muelleri*, *X. borealis*, and *X. clivii*; and the *fraseri* group includes *X. fraseri* and *X. pygmaeus* [14,21]. It has been proposed that the seven extant octoploid species arose from three distinct allopolyploidization events [22]. Thus, *X. lenduensis* and *X. vestitus* share a common tetraploid ancestor; *X. amieti*, *X. andrei*, and *X. boumbaensis* form a second group; and *X. itombwensis* and *X.* *wittei* constitute a third group. Further allopolyploidization events involving a tetraploid species and an octoploid species within the second group have given rise to the dodecaploid species *X. longipes* and *X. ruwenzoriensis*.

The tropical clawed frog *Silurana tropicalis* retains the diploid status (chromosome number 2n = 20) that is thought to be related to the ancestral state but putative allopolyploidization events within the *Silurana* lineage have given rise to the Cameroon clawed frog *S. epitropicalis* with chromosome number 2n = 40 as well as at least two further unnamed tetraploid species [12,23]. The monotypic genus *Pseudhymenochiru*s is accepted as sister group to genus *Hymenochirus* (African dwarf frogs) which includes four described species [12]. No allopolyploidization and higher level of ploidy have been reported for species belonging to these two genera [17]. 

## 3. Peptides with Antimicrobial Activity

Although peptides with hemolytic activity had been identified in skin secretions of frogs from the genera *Bombina* and *Rana* earlier, *X. laevis* was the first amphibian species in whose skin peptides with antimicrobial activity (magainin-1 and -2) were unambiguously identified [24]. Subsequent analysis of *X. laevis* skin secretions has led to the isolation and characterization of peptide glycine-leucine amide (PGLa) and additional antimicrobial peptides with varying potencies and specificities that are derived from the post-translational processing of the biosynthetic precursors of caerulein and xenopsin [25,26]. These peptides have been termed caerulein precursor fragment (CPF) and xenopsin precursor fragment (XPF). A comparison of the amino acid sequences of procaerulein, promagainin, and proxenopsin, deduced from the nucleotide sequences of cDNAs, reveals significant structural similarity in the N-terminal regions of the precursors suggesting that the peptides may have evolved from a common ancestral gene by a series of duplication events [27]. Orthologs of magainin-1 and -2, PGLa, and CPF, and XPF have been identified in skin secretions of range of frog species belonging to the genus *Xenopus* (*X. amieti* [28], *X. andrei* [29], *X. borealis* [30], *X. clivii* [31], *X. lenduensis* [32], *X. muelleri* and an incompletely characterized species from West Africa referred to as “*Xenopus* new tetraploid 1” and provisionally designated *X. muelleri* West [33], *X. petersii* [32], *X. pygmaeus* [32], and *X. victorianus* [34]). Host-defense peptides have also been isolated from laboratory-generated F1 hybrids of *X. laevis ×* *X. muelleri* [35] and *X. laevis* *×* *X. borealis* [36]. Evolutionary pressure to conserve the primary structures of the antimicrobial peptides from *Xenopus* species has not been strong and the sequences of the procaerulein- and proxenopsin-derived peptides are particularly variable.

Peptides that belong to the PGLa family (PGLa-ST1, originally designated XT-5), the CPF family (CPF-ST1, -ST2, and -ST3, originally designated XT-1, XT-6, and XT-7), and the XPF family (XPF-ST1, -ST2, and -ST3 originally designated XT-2, XT-3, and XT-4) have been isolated from skin secretions of the diploid frog *S. tropicalis* [37]. Although a magainin peptide was not identified in *S. tropicalis* skin secretions, a search of the *S. tropicalis* genome database reveals the presence of a gene encoding a magainin-related peptide (referred to in this article as magainin-ST1) [38]. Peptides belonging to the magainin family (magainin-SE1), the PGLa family (PGLa-SE1 and -SE2), the CPF family (CPF-SE1, -SE2 and -SE3), and the X PF family (XPF-SE1, SE-2, SE-3 and -SE4), have been isolated from skin secretions of the tetraploid from *S. epitropicalis* [39].

More recently, peptidomic analysis of norepinephrine-stimulated skin secretions from the Congo dwarf clawed frog *Hymenochirus boettgeri* [40] and Merlin’s clawed frog *Pseudhymenochirus merlini* [41] has led to identification of a family of structurally related host-defense peptides, termed the hymenochirins, with broad-spectrum antimicrobial activity. The hymenochirins show very low structural similarity with the antimicrobial peptides isolated from skin secretions of *Silurana* and *Xenopus* species consistent with the proposed ancient divergence of the Xenopodinae and the sister-group genera *Hymenochirus* and *Pseudhymenochirus* [17,18]. The strongly conserved hymenochirins from *P. merlini* show closest structural similarity to hymenochirin-1 and hymenochirin-5 from *H. boettgeri*. Peptides with novel structural features and broad spectrum antimicrobial activity, termed pseudhymenochirin-1Pa, -1Pb, and -2Pa, were also isolated from *P. merlini* secretions [41]. Unexpectedly, skin secretions from those frogs from the genus *Pipa* examined to date (*Pipa pipa* and *Pipa parva*) do not appear to contain cytotoxic peptides (unpublished data).

### 3.1. Magainins

**Figure 1:**
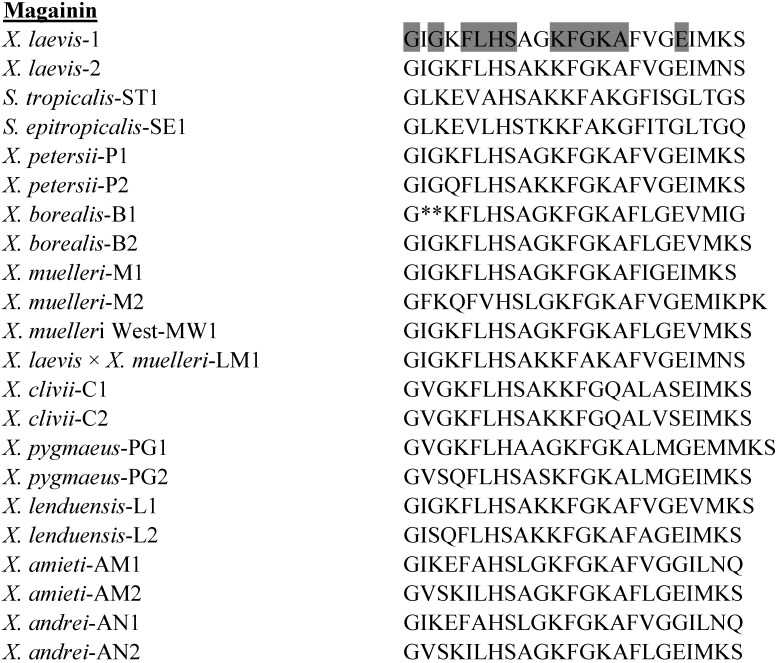
Primary structures of the magainin peptides isolated from skin secretions of frogs belonging to the genera *Xenopus* and *Silurana*. The amino acid sequence of magainin-ST1 was deduced from the corresponding nucleotide sequence of genomic DNA. In order to maximize structural similarity, gaps denoted by * have been introduced into some sequences. Strongly conserved residues are shaded.

The primary structures of the magainin peptides isolated to date from species in the genera *Xenopus* and *Silurana* are shown in Figure 1. Although probably the most intensively studied of all frog skin host-defense peptides, the magainins from the South African clawed frog *X. laevis* have only low or moderate antimicrobial potency against microorganisms. The hemolytic activity against human erythrocytes of magainin-2 in phosphate-buffered saline is low (the concentration producing 50% hemolysis, LC_50_ > 100 µM) but the peptide is strongly hemolytic when tested in 1 mM potassium phosphate buffer supplemented with 287 mM glucose (LC_50_ = 7 µM) [42]. Several recent studies have investigated in detail the mechanism of action by which magainin-2 produces bacterial cell death [43,44,45].

A large number of analogs of magainin-2 have been synthesized with a view to increasing antimicrobial potency while decreasing hemolytic activity [46,47]. These include hybrid peptides comprising fragments of magainin-2 coupled to fragments of other antimicrobial peptides such as cecropin A(1-8)-magainin-2(1-12) which displays strong antimicrobial activity against a range of antibiotic resistant bacterial and fungal strains and low hemolytic activity [48]. Adopting the strategy of increasing cationicity to promote antimicrobial potency has led to the development of the analogue, pexiganan (MSI-78) which represents an analogue of magainin-2 that contains an additional five lysyl residues and an α-amidated C-terminus [49]. It was developed initially as a topical anti-infective agent for the treatment of infected foot ulcers in diabetic patients and as a possible treatment for impetigo. Pexiganan showed broad-spectrum antibacterial activity when tested against 3,109 clinical isolates of Gram-positive and Gram-negative aerobic and anaerobic bacteria [50]. The minimum inhibitory concentration (MIC) at which 90% of isolates were inhibited (MIC_90_) was less than or equal to 32 µg/mL for several pathogens that are commonly recovered from diabetic foot wounds, including *Staphylococcus* spp., *Streptococcus* spp., *Corynebacterium* spp., *Pseudomonas* spp., *Acinetobacter* spp., *Bacteriodes* spp., *Peptostreptococcus* spp., and *Escherichia coli*. For 92% of the isolates tested, minimum bactericidal concentration (MBC) was the same or within a twofold difference of the MIC, consistent with a bactericidal action. A related study involving 2,515 bacterial isolates from infected foot ulcers from diabetic patients produced similar results with MIC_90_ values for pexiganan of 16 µg/mL or less for a range of Gram-positive aerobes, Gram-negative aerobes and facultative anaerobes [51]. *Proteus* spp. and *Serratia* spp. are also known to colonize foot ulcers but, in common with most frog skin peptides, pexiganan was inactive against strains of *Proteus mirabilis* and *Serratia marcescens*. In phase III multicentre, randomised, double-blind trials in diabetic patients with infected foot ulcers, topical application of pexiganan acetate (1%) achieved clinical cure or improvement in about 90% of patients, a success rate comparable to oral ofloxacin (800 mg/day) used in the control group [52]. The study indicated that the agent was well tolerated. However, the Food and Drug Administration did not approve marketing of this agent on the grounds that efficacy has not been sufficiently demonstrated.

Other examples of activity of magainin-2 and its analogues against important human pathogens include *Helicobacter pylori* [53], *Salmonella typhimurium* [54], the anaerobic periodontal pathogens, *Porphyromonas gingivalis*, *Fusobacterium nucleatum*, and *Prevotella loeschei* [55], and *Acanthamoeba polyphagia*, a protozooan responsible for ocular infection in contact lens wearers [56].

### 3.2. Peptide Glycine-Leucine-Amide (PGLa) Peptides

**Figure 2:**
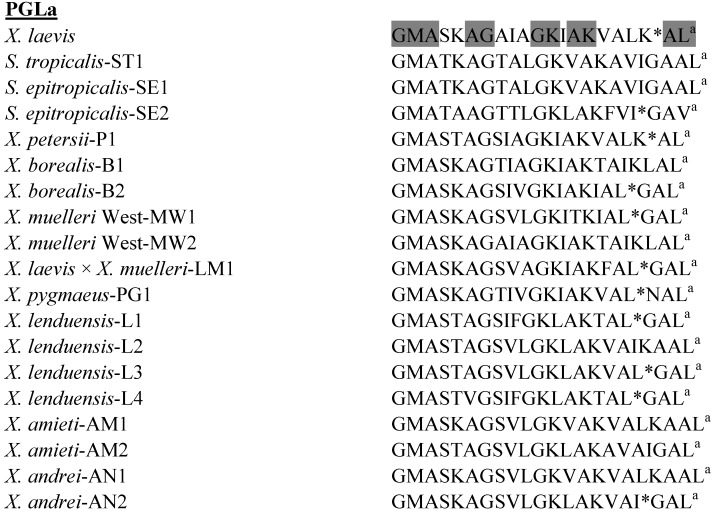
Primary structures of the peptide glycine-leucine-amide (PGLa) peptides isolated from skin secretions of frogs belonging to the genera *Xenopus* and *Silurana*. ^a^ denotes C-terminal α-amidation. In order to maximize structural similarity, gaps denoted by * have been introduced into some sequences. Strongly conserved residues are shaded.

The primary structures of the PGLa peptides isolated to date from species in the genera *Xenopus* and *Silurana* are shown in Figure 2. Although PGLa from *X. laevis* has often been used as a model peptide to study membrane-peptide interactions, its therapeutic potential as an anti-infective agent has not been extensively investigated. PGLa from *X. laevis* is active against amphotericin B-resistant *Candida albicans*, *Candida krusei*, and *Aspergillus fumigatus* strains and against a fluconazole-resistant *Candida glabrata* isolate [57]. PGLa acts synergistically with magainin-2 both in killing *E. coli* and permeabilizing protein-free liposomes so that the peptides are much more potent when added together than when added alone [58]. The mechanism of action of the peptide alone [59] and in combination with magainin-2 [60] has been studied in detail.

PGLa-AM1 from *X. amieti* shows broad spectrum bactericidal activity with MIC values ≤ 25 μM against reference strains of *E. coli* and *S. aureus* combined with very low toxicity to human red blood cells (LC_50_ > 500 μM) [28]. PGLa-AM1 shows potent growth-inhibitory activity against clinical isolates of antibiotic-resistant *A. baumannii*, including strains that are resistant to colistin (MIC in the range 4–32 μM) [61]. The peptide is also active against multiple clinical isolates of antibiotic-resistant *S. maltophilia* (MIC in the range 2–16 μM) (unpublished data).

PGLa-AM1 from *X. amieti* showed potent growth-inhibitory activity against reference stains of both Gram positive (*Streptococcus mutans* MIC = 1.2 µM) and Gram negative (*F*. *nucleatum* MIC = 1.5 µM) oral bacteria that are associated with tooth decay and periodontal disease. When tested against the opportunistic yeast pathogen *C. albicans*, PGLa-AM1 also proved to be highly effective (MIC = 7.5 µM). PGLa-AM1 showed no cytotoxicity to primary dental pulp fibroblasts at concentrations up to 10 µM and did not stimulate production of the proinflammatory cytokine IL-8 (unpublished data).

### 3.3. Caerulein Precursor Fragment (CPF) Peptides

**Figure 3:**
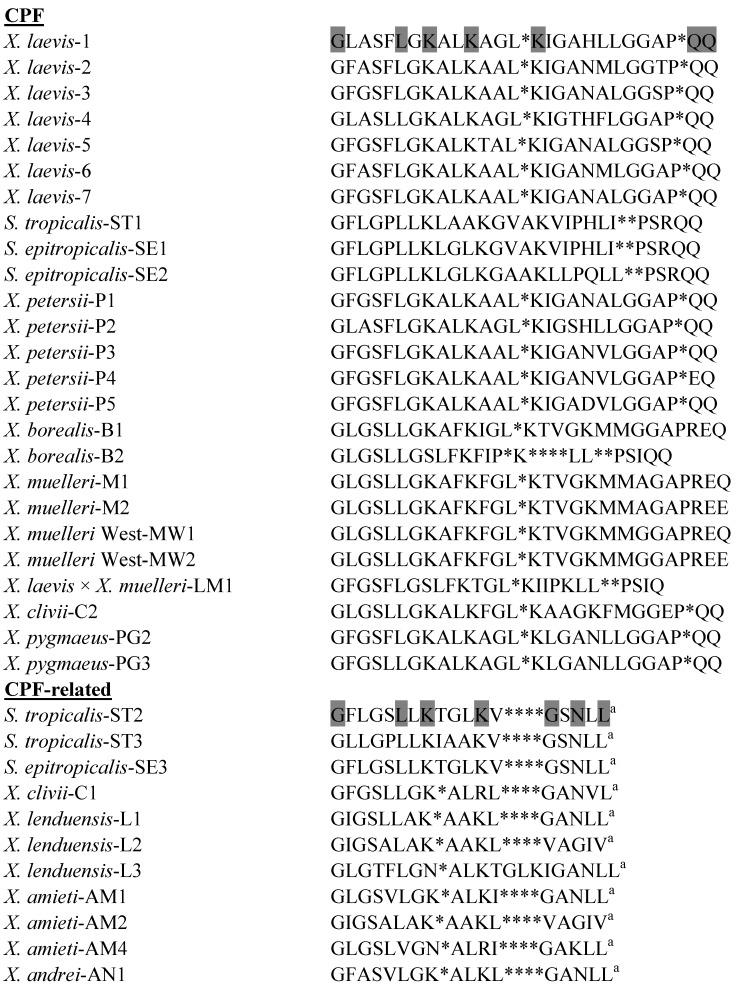
Primary structures of the caerulein precursor fragment (CPF) peptides isolated from skin secretions of frogs belonging to the genera *Xenopus* and *Silurana*. ^a^ denotes C-terminal α-amidation. In order to maximize structural similarity, gaps denoted by * have been introduced into some sequences. Strongly conserved residues are shaded.

The primary structures of the CPF peptides and CPF-related peptides isolated to date from species in the genera *Xenopus* and *Silurana* are shown in Figure 3. CPF-C1, the most abundant antimicrobial peptide in skin secretions of *X. clivii*, inhibits the growth of the Gram-negative bacteria *E.* *coli*, *A. baumannii*, *K. pneumoniae*, and *P. aeruginosa* (MIC in the range 3–25 µM), suggesting potential for development into an anti-infective agent for use against these emerging antibiotic-resistant pathogens [31]. CPF-AM1 from *X. amieti* shows broad spectrum bactericidal activity with MIC values ≤ 25 μM against reference strains of *E. coli* and *S. aureus* combined with moderate toxicity to human red blood cell (LC_50_ = 150 μM) [28]. The peptide shows potent growth-inhibitory activity against clinical isolates of multidrug-resistant *A. baumannii*, including strains that are resistant to colistin (MIC in the range 2–8 μM) [61]. CPF-AM1 is also active against multiple clinical isolates of antibiotic-resistant *S. maltophilia* (MIC in the range 2–8 μM) (unpublished data). Like PGLa-AM1, CPF-AM1 showed potent growth inhibitory activity against reference strains of a range of microorganisms associated with the oral cavity, such as *S. mutans* (MIC = 2.5 µM), *Lactobacillus* *acidophilus* (MIC = 2.5 µM), *F. nucleatum* (MIC = 2.2 µM), and *C. albicans* (MIC = 9.9 µM) and was not cytotoxic to primary dental pulp fibroblasts at concentrations up to 10 µM (unpublished data).

The CPF-related peptide, CPF-ST3 from *S. tropicalis* (formerly described as peptide XT-7 [37]), shows potent broad spectrum antimicrobial activity but is moderately hemolytic against human erythrocytes (LC_50_ = 140 μM) thus limiting its therapeutic applicability. However, the analog [G4K] CPF-ST3 is non-hemolytic (LC_50_ > 500 μM) and retains potent antimicrobial activity [62]. Proton NMR spectroscopy has demonstrated that the reduced toxicity of the analog correlates with a decrease in helicity as well as an increase in cationicity [63]. CPF-SE2 (MIC = 2.5 µM) and CPF-SE3 (MIC = 5 µM) from *S. epitropicalis* show potent growth-inhibitory activity against a range of clinical isolates of MRSA but their utility as systemic anti-infective drugs is again limited by appreciable hemolytic activity against human erythrocytes for CPF-SE2 (LC_50_ = 50 µM) and moderate activity for CPF-SE3 (LC_50_ = 220 µM) [39]. Nevertheless, the peptides may find application as topical agents in treatment of MRSA skin infections and decolonization of MRSA carriers.

### 3.4. Xenopsin Precursor Fragment (XPF) Peptides

**Figure 4:**
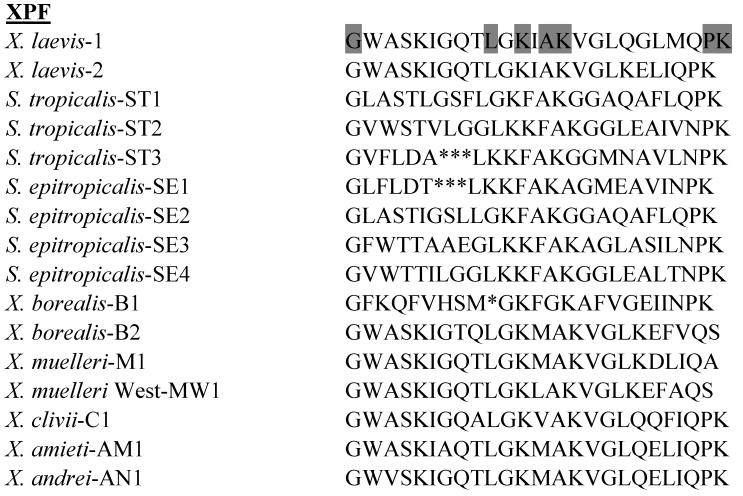
Primary structures of the xenopsin precursor fragment (XPF) peptides isolated from skin secretions of frogs belonging to the genera *Xenopus* and *Silurana*. In order to maximize structural similarity, gaps denoted by * have been introduced into some sequences. Strongly conserved residues are shaded.

The primary structures of the XPF peptides isolated to date from species in the genera *Xenopus* and *Silurana* are shown in Figure 4. XPF peptides are widely distributed in skin secretions of clawed frogs and have also been identified in the gastrointestinal tract of *X. laevis* [64]. However, the antimicrobial potencies of XPF peptides are generally lower than those of CPF peptides and their potential for development into anti-infective agents has not been well studied. Of those XPF peptides studied to date, XPF-C1 from *X. clivii* shows relatively high growth-inhibitory potency against *E. coli* (MIC = 12.5 µM) but was inactive against *S. aureus* [31].

### 3.5. Hymenochirins

**Figure 5:**
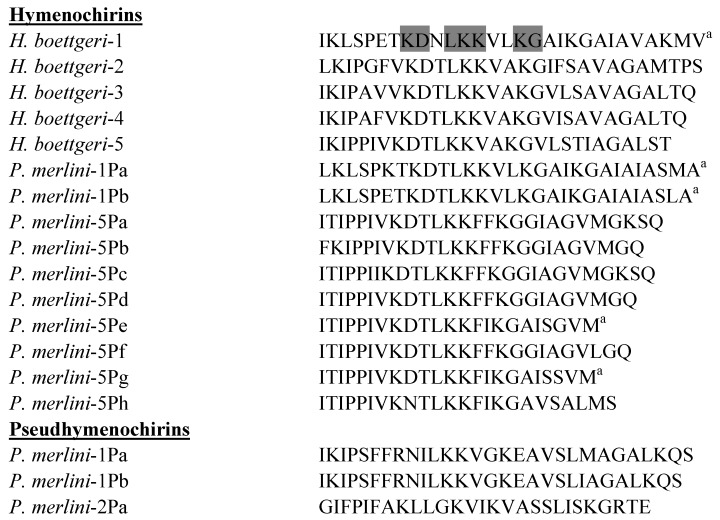
Primary structures of the hymenochirins isolated from skin secretions of the frogs *Hymenochirus* *boettgeri* and *Pseudhymenochirus merlini*, and the pseudhymenochirins from *P. merlini.* ^a^ denotes C-terminal α-amidation. Strongly conserved residues are shaded.

The primary structures of the hymenochirins isolated to date from *H. boettgeri* and *P. merlini* are shown in Figure 5. Hymenochirin-1B was first isolated from norepinephrine-stimulated skin secretions from the Congo dwarf clawed frog *H. boettgeri* [40]. The peptide is cationic (molecular charge = +6 at pH 7) and has the propensity to adopt an amphipathic α-helical conformation in a membrane-mimetic environment. Hymenochirin-1B displays moderate growth-inhibitory activity against reference strains of Gram-negative (*E. coli* MIC = 25 µM) and Gram-positive bacteria (*S. aureus* MIC = 12.5 µM) and its hemolytic activity against human erythrocytes is relatively low (LC_50_ = 213 µM). Analogs in which the Pro^5^, Glu^6^ and Asp^9^ on the hydrophilic face of the α-helix are substituted by one or more L-lysine residues show increased antimicrobial potency (up to 8-fold) but the peptides are more hemolytic. Increasing the cationicity of hymenochirin-1B while reducing helicity by substitutions with D-lysine generates analogs that are between 2- and 8-fold more potent than the native peptide and are equally or less hemolytic. [E6k,D9k]hymenochirin-1B represents a candidate for drug development as it shows high potency against clinical isolates of MRSA and a range of Gram-negative bacteria, including multidrug-resistant strains of *A. baumannii* and *S. maltophilia* (MIC in the range 0.8–3.1 µM) and New Dehli Metallo-β-Lactamase-1 (NDM-1)-producing clinical isolates of *K. pneumoniae*, *E. coli*, *Enterobacter cloacae* and *Citrobacter freundii* (MIC in the range 3.1–6.25 µM), and low hemolytic activity (LC_50_ = 302 µM) [65].

Close upPreliminary data indicate that hymenochirin-1Pa, pseudhymenochirin-1Pb, and pseudhymenochirin-2Pa from *P. merlini* also show potent growth-inhibitory potency against multidrug-resistant clinical isolates of *S. aureus*, *Staphylococcus epidermidis*, *A. baumannii*, and *S. maltophilia* but are more hemolytic than hymenochirin-1B (unpublished data).

## 4. Peptides with Anti-Cancer Activity

The problems posed by the emergence of multidrug resistance in the treatment of bacterial infections are also encountered in cancer chemotherapy. Because of their non-specific and destructive mechanism of action, cell-penetrating peptides show therapeutic potential for development into anti-cancer agents in cases where the tumor is not responsive to conventional pharmaceutical therapy. In addition, certain cationic antimicrobial peptides can produce tumor cell death by instigating apoptosis via mitochondrial membranes disruption and act as anti-angiogenic factors [66]. Analogs of naturally occurring frog skin host-defense peptides, including those from species within the family Pipidae, have been developed that show selective cytotoxicity against tumor cells and so have potential for development into anti-cancer agents.

Magainin-2 and its C-terminally α-amidated, carboxypeptidase-resistant analog, magainin G show potential as anticancer agents displaying tumoricidal activity against human small cell lung cancer cell lines [67], the RT4, 647V, and 486P bladder cancer cell lines [68], and against suspension cultures of a wide range of hematopoietic cell lines [69]. Out of a range of antimicrobial peptides tested, the magainin-2 analog, pexiganan shows the greatest cytotoxic activity against the U937 human histiocytic lymphoma cell line [70]. The anti-tumor activity of protease-resistant all D-amino acid magainin-2 amide (MSI-238) is markedly superior to the parent compound displaying high potency *in vitro* against non-small cell lung adenocarcinoma A549 cells and *in vivo* against P388 leukemia, S180 ascites, and a spontaneous ovarian tumor [71]. The cytotoxic mechanism of [F5W]magainin-2 against HeLa cells, has been investigated and involves initial interaction of the peptide with cell surface gangliosides [72].

Hymenochirin-1B shows high cytotoxic potency against A549 cells (LC_50_ = 2.5 µM), breast adenocarcinoma MDA-MB-231 cells (LC_50_ = 9.0 µM), colorectal adenocarcinoma HT-29 cells (LC_50_ = 9.7 µM), and hepatocarcinoma HepG2 cells (LC_50_ = 22.5 µM) with appreciably less hemolytic activity against human erythrocytes (LC_50_ = 213 µM) [73]. Structure-activity relationships were investigated by synthesizing analogs of hymenochirin-1B in which Pro^5^, Glu^6^ and Asp^9^ on the hydrophilic face of the peptide helix are replaced by one or more l-lysine or d-lysine residues. The [D9K] analog displays the greatest increase in potency against all four cell lines (up to 6-fold) but hemolytic activity also increases (LC_50_ = 174 µM). The [D9k] and [E6k,D9k] analogs retain relatively high cytotoxic potency against the four tumor cell lines (LC_50_ in the range 2.1–21 µM) but show reduced hemolytic activity (LC_50_ > 300 µM).

CPF-ST3 (peptide XT-7) from *S. tropicalis* shows only moderate cytotoxic potency against HepG2 cells (LC_50_ = 75 µM) but increasing the cationicity of the peptide by appropriate amino acid substitutions by l-lysine that preserve amphipathicity results in a progressive increase in activity ([S15K] CPF-ST3, LC_50_ = 24 µM; ([S15K,N16K]CPF-ST3, LC_50_ = 10 µM; ([P5K,S15K,N16K] CPF-ST3, LC_50_ = 5 µM) [62].

## 5. Peptides with Anti-Viral Activity

Viruses cannot reproduce independently and instead use host cells for replication. Finding targets for an antiviral drug that would interfere specifically with the virus without harming the host cells poses a challenge for designing of safe and effective antivirals. Viral life cycles vary in their precise details depending on the species of virus but all share a general pattern: binding to a specific receptor on the surface of the host cell, uncoating of the virus inside the cell to release its genome, replication using host-cells machinery, assembly of virus progeny and release of viral particles to infect new host cells. Viruses that have a lipid envelope must also fuse their envelope with the target cell, or with a vesicle that transports them into the cell, before they can uncoat. Certain peptides that are present in frog skin secretions have demonstrated potent antiviral activity, either by directly inactivating the virus particles or by interfering with the initial steps of the viral reproductive cycle such as binding to specific cell surface receptors and subsequent entry into the cytoplasm. These properties, combined the short contact time required to induce killing, have led to their consideration as candidates for development into novel antiviral agents.

Magainin-1 and -2 from *X. laevis* show antiviral properties against herpes simplex virus type 1 (HSV-1) and herpes simplex virus type 2 (HSV-2) but were inactive against the arenavirus, Junin virus The peptides do not appear to inactivate the HSV particles directly but rather target important steps in the viral reproductive cycle [74]. Magainin-2 and PGLa from *X. laevis* markedly reduced the infectivity of channel catfish virus but were less potent against frog virus 3 [75]. Magainin-1 was ineffective against both viruses. Mechanistic studies have shown that an Ala-substituted magainin-2 amide analog directly inactivates vaccinia virus by disrupting and removing the outer membrane envelope [76].

Both CPF-AM1 and PGLa-AM1 from *X. amieti* are capable of destroying more than 90% of extracellular HSV-1 virions within the first 5 min of direct contact (unpublished data). In addition, these two peptides inhibit the viral penetration and replication in Madin-Darby Bovine Kidney (MDBK) cells when applied at non-toxic concentrations (≤200 µM). Similarly, CPF-ST3 (peptide XT-7) can destabilize HSV-1 particles and block virus entry and/or replication with EC_50_ = 87 µM (unpublished data). For additional amphibian peptides with anti-bacterial, anti-viral and anti-cancer activities, interested readers may refer to the antimicrobial peptide database at http://aps.unmc.edu/AP.

## 6. Conclusions

The antimicrobial and hemolytic activities of the peptides showing the greatest potential for development into therapeutically valuable anti-infective agents are summarized in Table 1. Over 25 years have passed since the discovery of the magainins in the skin of the African clawed frog, *X. laevis.* Despite displaying potent activity against strains of antibiotic-resistant bacteria and against certain pathogenic fungi and protozoa, the therapeutic potential of frog skin antimicrobial peptides has yet to be realized. Currently, no anti-infective peptide based upon their structures has been adopted in clinical practice. Consequently, interest is moving away from their use as antimicrobials towards other potential clinical applications.

**Table 1:** Minimum Inhibitory Concentrations (µM) and hemolytic activities against human erythrocytes (µM) of the peptides with greatest therapeutic potential against reference strains and clinical isolates of clinically relevant microorganisms.

Peptide	*E. coli* ATCC 25726	*S. aureus* ATCC 25923	MRSA isolates	*A. baumannii* isolates	*S. maltophilia* isolates	*K. pneumoniae* isolates	LC_50_
[G4K]CPF-ST3	12.5	6.25	ND	1.6–3.1	ND	ND	>500
PGLa-AM1	12.5	25	ND	4–32	2–16	ND	>500
CPF-AM1	12.5	6.25	ND	2–8	2–8	25	150
CPF-C1	6.25	6.25	ND	3.1	ND	25	140
CPF-SE2	40	2.5	2.5	ND	ND	ND	50
CPF-SE3	40	2.5	5	ND	ND	ND	220
[E6k,D9k] hymenochirin-1B	3.1	1.6	3.1–6.25	1.6	0.8–3.1	3.1–6.25	300

ND: not determined.

Several frog peptides that were first identified on the basis of their abilities to inhibit growth of bacteria have been shown to stimulate release of insulin from BRIN-BD11clonal β-cells and improve glucose tolerance in mice and so show potential for treatment of patients with Type 2 diabetes (reviewed in [77]). For example, CPF-1, CPF-3, CPF-5 and CPF-6 from *X. laevis* and CPF-SE1 from *S. epitropicalis* produced a significant increase in the rate of insulin release from BRIN-BD11 cells at concentrations as low as 0.03 nM. Similarly, magainin-AM1, magainin-AM2, CPF-AM1, and PGLa-AM1 stimulated release of the incretin peptide, GLP-1 from GLUTag cells with magainin-AM2 exhibiting the greatest potency (minimum concentration producing a significant stimulation = 1 nM) and CPF-AM1 producing the maximum stimulatory response (3.2-fold of basal rate at a concentration of 3 µM) [78].

Several frog skin peptides with cytotoxic properties have subsequently been shown to possess complex cytokine-mediated immunomodulatory activities. Effects on the production of both pro-inflammatory and anti-inflammatory cytokines have been observed (reviewed in [79]). Endotoxemic complications, such as severe sepsis and septic shock, following infection by Gram-negative bacteria are caused by release of lipopolysaccharide from bacterial membrane into the bloodstream and result in high levels of mortality. The importance of agents that modulate the immune function of the host in the treatment of sepsis is recognized [80]. The [E6k,D9k] analog of hymenochirin-1B increases the production of anti-inflammatory cytokine IL-10 from both unstimulated and concanavalin A-stimulated human peripheral blood mononuclear cells without increasing the rate of production of the pro-inflammatory cytokines TNF-α and IL-17 suggesting a possible therapeutic role in attenuating the inflammatory response triggered by bacteria [65].
